# Performance of fine particulate matter data on air quality in an epidemiological study in Salvador, Brazil

**DOI:** 10.1590/1980-549720240068

**Published:** 2024-12-16

**Authors:** Ludmilla Viana Jacobson, Sandra Hacon, Vanúcia Schumacher, Clarcson Plácido Conceição Dos Santos, Nelzair Vianna

**Affiliations:** I Universidade Federal Fluminense, Department of Statistics – Niterói (RJ), Brazil.; II Fundação Oswaldo Cruz, Sérgio Arouca National School of Public Health – Rio de Janeiro (RJ), Brazil.; III Instituto Nacional de Pesquisas Espaciais, Center for Weather Forecasting and Climate Studies, São José dos Campos (SP), Brazil.; IV Escola Bahiana de Medicina e Saúde Pública, Graduate Program in Medicine and Human Health – Salvador (BA), Brazil.; V Fundação Oswaldo Cruz, Laboratory of Molecular Biology and Biostatistics – Salvador (BA), Brazil.

**Keywords:** Air pollution, Public health, Time series, Information systems

## Abstract

**Objective:**

To evaluate the performance of satellite-derived PM2.5 concentrations against ground-based measurements in the municipality of Salvador (state of Bahia, Brazil) and the implications of these estimations for the associations of PM2.5 with daily non-accidental mortality.

**Methods:**

This is a daily time series study covering the period from 2011 to 2016. A correction factor to improve the alignment between the two data sources was proposed. Effects of PM2.5 were estimated in Poisson generalized additive models, combined with a distributed lag approach.

**Results:**

According to the results, satellite data underestimated the PM2.5 levels compared to ground measurements. However, the application of a correction factor improved the alignment between satellite and ground-based data. We found no significant differences between the estimated relative risks based on the corrected satellite data and those based on ground measurements.

**Conclusion:**

In this study we highlight the importance of validating satellite-modeled PM2.5 data to assess and understand health impacts. The development of models using remote sensing to estimate PM2.5 allows the quantification of health risks arising from the exposure.

## INTRODUCTION

Air pollution is an environmental threat with multiple impacts on human health. The World Health Organization (WHO) draws attention to the burden of disease associated with air pollution. Millions of premature deaths from cerebrovascular accident, heart diseases, lung cancer, and chronic and acute respiratory diseases, including asthma, could be prevented by reducing air pollution levels^1^. Vulnerable populations, including children, older adults, pregnant women, and people with chronic diseases, are at greater risk^2–4^. In Brazil, the Pan American Health Organization reported 51 thousand annual deaths associated with air pollution^5,6^.

Exposure to particulate matter with a diameter of less than 2.5 μm (PM2.5) has been investigated in many studies and has been shown to be a robust indicator of health risk associated with different emission sources^7,8^. It is worth noting that PM2.5 includes the inhalable particulate matter PM10. In urban areas of developed countries, the fraction of PM2.5 to PM10 ranged from 0.5 to 0.8^9^. In the study carried out by Cohen et al.^10^ in several urban areas of the world, the fraction of 0.5xPM10 was adopted to estimate PM2.5 and calculate the number of premature deaths associated with exposure to the pollutant. In Brazil, in a study carried out in an urban area, the average PM2.5/PM10 ratio was 0.52^11^. Both PM2.5 and PM10 can be inhaled, but PM2.5 has a greater potential health risk, as fine particles can penetrate deep into the respiratory tract and reach the lungs^12^.

Time series epidemiological studies have been used to quantify the risks associated with exposure to air pollutants and health outcomes^13–18^. One of the relevant challenges in investigating the effects of air pollution on the health of the population in the main capitals of Brazil is the lack of continuous monitoring of atmospheric pollutants. In Brazil, the air quality monitoring network is restricted, which represents a limitation for the development of epidemiological studies. Of the 26 Brazilian states, 11 have an air quality monitoring system, 80% of which are located in the Southeast Region, but they present incomplete information and limited access regarding air quality^19^. For time series epidemiological studies, the study period and the availability of air quality data is an important factor in reducing uncertainties, although proxy indicators and information from predictive models can also be used as long as their limitations are discussed^20–22^.

The National Institute for Space Research (*Instituto Nacional de Pesquisas Espaciais* – INPE), in collaboration with other Brazilian institutions, developed the Environmental Information System Integrated with Environmental Health (*Sistema de Informações Ambientais Integrado à Saúde Ambiental* – SISAM) with the aim of assisting health programs on the impacts of atmospheric pollutant emissions on human health. SISAM is an *online* platform that provides the concentrations of atmospheric pollutants, estimated by remote sensing with a spatial resolution of approximately 12.5 km, which allows a spatiotemporal characterization of the variability of daily exposure for all municipalities in Brazil, at no cost to the user^23^.

Our objective was to evaluate the performance of PM2.5 estimates from the SISAM platform in comparison with ground-based measurements in the municipality of Salvador, in the state of Bahia, Brazil, as well as their implications for the associations of PM2.5 with non-accidental mortality.

## METHODS

### Study design and area

This is an ecological study of daily time series, from 2011 to 2016.

The research was carried out in Salvador, the second largest city in northeastern Brazil and the fifth most populous in the country, with a population of approximately 2.42 million according to the 2022 Census^24^. The municipality is divided into ten city councils-neighborhoods and these are subdivided into 163 neighborhoods.

**Figure 1:**
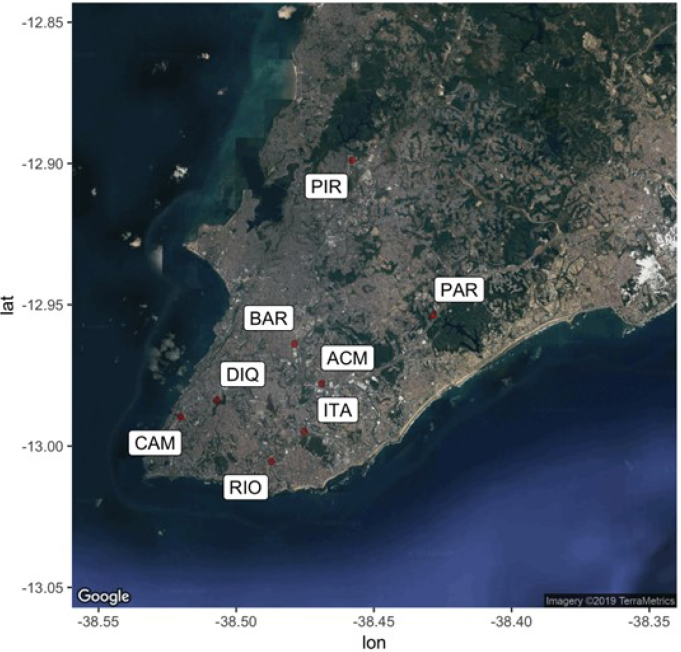
Location of fixed monitoring stations in Salvador (state of Bahia – BA), 2011 to 2016.

PIR: Pirajá; PAR: CAB/Paralela station; CAM: Campo Grande; RIO: Vermelho River; DIQ: Tororo Dam; ITA: Itaigara; BAR: Barros Reis; ACM: Av. ACM/Detran.

From 2011 to 2016, air quality monitoring in Salvador was managed by Cetrel, a private Brazilian company specialized in environmental monitoring. During this period, Salvador's air quality monitoring network consisted of eight fixed monitoring stations, strategically located throughout the city. These stations provided data with one-hour temporal resolution on levels of pollutants such as PM10, SO_2_, CO, O_3_, and NO_2_; as well as meteorological parameters such as wind speed and direction, temperature, humidity, and rainfall. Among the monitoring stations, five were in operation as of 2011, namely: Campo Grande, Dique do Tororó [Tororó Dam], CAB/Dique do Paralela [Paralela Dam], Pirajá, and Rio Vermelho [Vermelho River]. The operation of the three remaining stations — such as Av. ACM/Detran, Av. Barros Reis, and Itaigara — began in 2013. Until the beginning of this study, daily measurements of atmospheric pollutants available in Salvador were only related to the 2011-2016 period. The study area and specific location of monitoring stations are shown in Figure 1.

### Database

#### Measured data of air pollutants and meteorological parameters

PM10, temperature, and humidity data were obtained from Cetrel monitoring stations for the 2011-2016 period. In the absence of direct PM2.5 measurements, the ratio of 0.5 for PM2.5/PM10 was used, commonly accepted for urban areas and as recommended by Ostro^25^. This approach allowed estimating PM2.5 concentrations based on recorded PM10 levels.

#### Environmental Information System Integrated with Environmental Health (SISAM) platform

The SISAM platform presents spreadsheets with meteorological data and air quality for all states and municipalities, by date and time^26^. SISAM is sourced from the Copernicus Atmosphere Monitoring Service (CAMS), a global reanalysis dataset of atmospheric composition produced by the European Center for Medium-Range Weather Forecasts with the updated Integrated Forecasting System. CAMS combines information from *in situ* and satellite observations with computer models of the atmosphere to generate the most accurate estimate possible of atmospheric gases and aerosols. CAMS validation is carried out periodically and coordinated by the Royal Dutch Meteorological Organization^27,28^. Innes et al.^29^ evaluated the performance of CAMS reanalyses by comparing previous versions, and Wang et al.^30^ validated CAMS using aircraft measurements in different parts of the world, including Brazil.

For the analyses of this study and the spatiotemporal characterization of the variability of daily exposure in Salvador, the daily average of PM2.5 from 2011 to 2016 was considered.

#### Data on deaths

The investigated health outcome was the total number of daily deaths from all non-accidental causes. The data on deaths were those made available in the Mortality Information System (*Sistema de Informação sobre Mortalidade* – SIM) through the Information Technology Department of the Brazilian Unified Health System. Accidental causes, those recorded in the death certificate as the underlying cause classified according to codes S00 to T98, V01 to Y98, Z00 to Z99, U00 to U99 (ICD10) were excluded from the analyses.

### Data analysis

#### Agreement measurements

The agreement between the daily PM2.5 data modeled from SISAM and the measured data from Cetrel monitoring stations was investigated using the t-test for paired samples, the Bland-Altman^31^ method, and the concordance correlation coefficient^32,33^. The paired t-test was used to assess differences between measurement methods regarding systematic bias. The Bland-Altman method consists of the scatter plot between the differences (Difference=PM2.5 SISAM – PM2.5 Measured) and the means [Mean=(PM2.5 SISAM–PM2.5 Measured)/2]. In this method, means are used as estimates of the actual PM2.5 value and, therefore, it is possible to investigate the relationship of errors with the actual PM2.5 value. The method also presents the limits of agreement estimated using the mean of the differences (bias) and their standard deviation (sd), as bias±1.96sd. The heteroscedasticity of the error was assessed by Pearson's correlation coefficient between the differences (Y) and the means (X). The concordance correlation coefficient (CCC) was used to assess how much the methods differ from each other.

The analyses were performed using the R 3.4.2.2 program^34^, *epiR* ^35^ and *blandr* ^36^ libraries. A 5% significance level was adopted in the interpretation of the results of the hypothesis tests.

#### Calibration of data from the Environmental Information System Integrated with Environmental Health (SISAM)

The daily time series of observed (Cetrel data) and modeled (SISAM data) PM2.5 were compared using graphs and descriptive statistics. Subsequently, the absolute difference indicator (Δ_*t*_=PM2.5observed_t_ – PM2.5modeled_t_) was calculated.

In data calibration, it was proposed to define an additive term based on the mean of the absolute difference indicator (Δ_*t*_), in the period from 2011 to 2016. The calibrated PM2.5 was calculated by the equation:


$$PM_{2.5}calibrated_{t}=PM_{2.5t}+\bar{x}$$

Where:

$\bar{x}=\frac{\sum_{t=1}^{n}\Delta_{t}}{n}$ , PM_2.5t_ is the PM2.5 modeled from SISAM on day t; and "n" is the total number of days in the period from 2011 to 2016.

This method was used to correct the bias observed in the SISAM data.

#### Statistical analysis

To evaluate the performance of SISAM data in epidemiological studies in Salvador, an ecological study of daily time series was proposed from 2011 to 2016. The health effects based on data measured by fixed monitors were compared with the effects estimated using calibrated SISAM data.

In the time series analysis, generalized additive models (GAM) were adjusted, considering the Poisson distribution with a logarithmic link function, combined with the distributed lag model^16,37,38^. The response variable was the daily count of the number of deaths, and the explanatory variables were the calendar day indicator for trend and seasonality adjustment, temperature, humidity, day of the week indicator, national holiday indicator, and PM2.5.

The temporal trend and seasonality were adjusted in the model by a thin plate spline, included in the regression with 4 degrees of freedom (df) per year; the daily mean temperature with a three-day lag (Lag 3) was adjusted by a thin plate spline with 2 df; the daily mean humidity of Lag 3 was adjusted by a thin plate spline with 2 df. Several adjustments were evaluated for the temporal trend and seasonality, varying the type of spline and degrees of freedom (df: 4 to 6 per year). Likewise for temperature and humidity, in which in addition to the daily means, the lags of Lag 1, Lag 2, and Lag 3 were evaluated. The choice of the number of df and the best fit for the model was based on the Akaike Information Criterion (AIC), on scatter plots of the deviance and periodogram residuals. After choosing the best adjustment for the trend and seasonality of weather, temperature, and humidity, the variables indicating day of the week and national holiday were included.

As the effects of air pollution on mortality may occur on the same day of exposure or on later days, PM2.5 was adjusted by a distributed lag polynomial function. The adjustment considered a second-degree polynomial and a lag of up to 30 days (lag 0-30). Moreover, single lag effects from zero to five days were also estimated. The results were presented as relative risk (RR) and percentage increase in the risk of death for each increase of 10 μg/m^3^ of PM2.5 as well as the respective 95% confidence intervals (95%CI). Furthermore, the exposure-lag-response curve of the effects of PM2.5 on mortality accumulated over 30 days of exposure was presented.

## RESULTS

**Figure 2:**
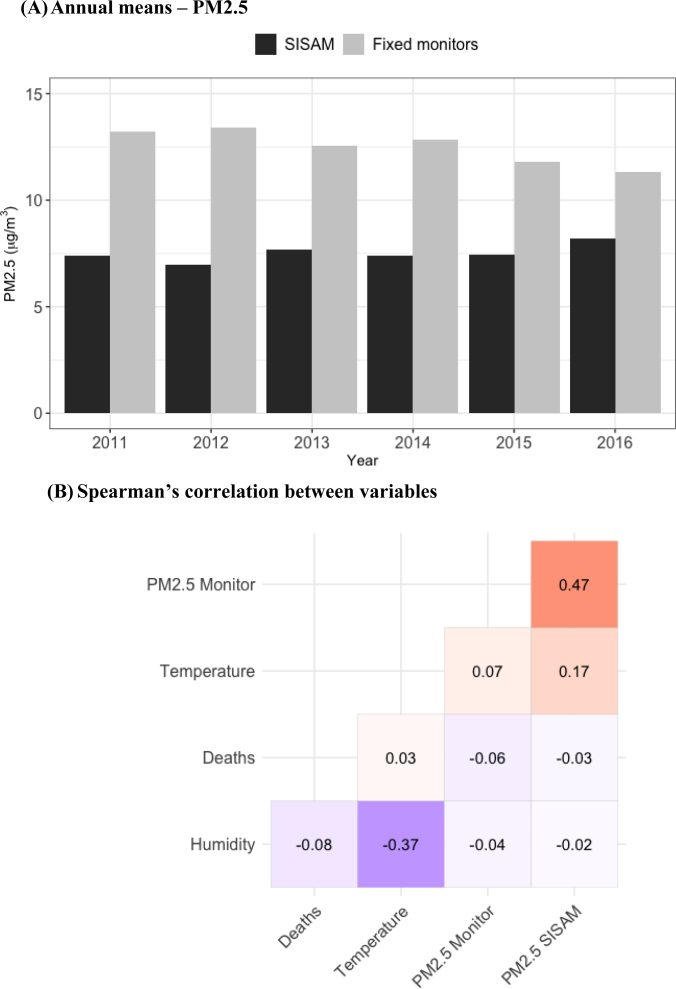
Annual means of data on particulate matter with a diameter of less than 2.5 μm (PM2.5) from the Environmental Information System Integrated with Environmental Health (SISAM) and monitoring stations (A) and correlations with meteorological variables (B). Salvador (BA), 2011 to 2016.

During the study period, the mean PM2.5 concentration, measured by Cetrel's fixed monitors and calculated based on measured PM10 data, was 12.5 μg/m^3^, while the modeled PM2.5 data from SISAM were, on average, 7.5 μg/m^3^±2.9 μg/m^3^. The annual means of PM2.5, shown in Figure 2A, exceeded the WHO recommendation for an annual mean air quality of 5 μg/m^3 39^. An average of 35±6.1 non-accidental deaths were recorded per day. Furthermore, the mean daily temperature was around 26°C, with relative humidity of 73% (Table 1). We found low correlations of environmental variables with non-accidental deaths (Figure 2B).

**Table 1:** Summary statistics of daily data on exposure, meteorological, and health variables. Salvador, 2011 – 2016.

	Daily measurements							
	Min.	P25	P50	Mean	P75	Max.	var	sd
PM2.5 (μg/m^3^) – SISAM	1.3	5.6	7.1	7.5	9.0	26.2	8.4	2.9
PM2.5 (μg/m^3^) – Fixed monitors	4.8	10.1	12.2	12.5	14.5	44.4	12.7	3.6
Temperature (°C)	22.0	25.3	26.4	26.3	27.4	29.3	1.9	1.4
Humidity (%)	59.1	69.1	72.2	73.0	76.3	93.5	31.2	5.6
Deaths from non-accidental causes	17.0	31.0	35.0	35.1	39.0	68.0	37.5	6.1

Min.: minimum value; Max.: maximum value; P25: 25th percentile; P50: 50th percentile; P75: 75th percentile; var: variance; sd: standard deviation.

**Figure 3:**
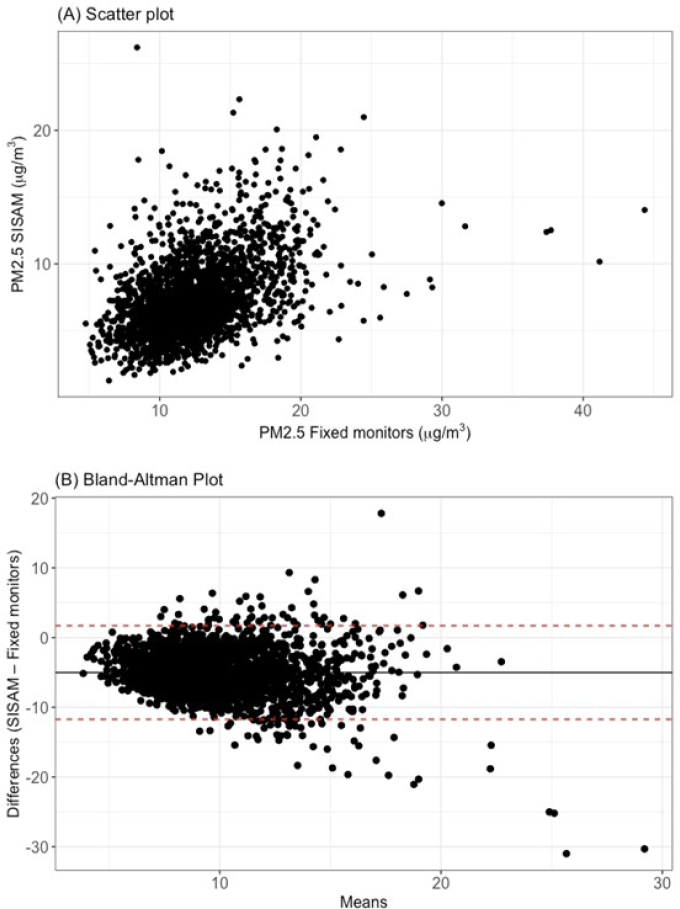
Comparison between modeled data on particulate matter with a diameter of less than 2.5 μm (PM2.5) (Environmental Information System Integrated with Environmental Health — SISAM) and data measured by monitoring stations (Fixed monitors). Salvador (BA), 2011 to 2016.

**Figure 4:**
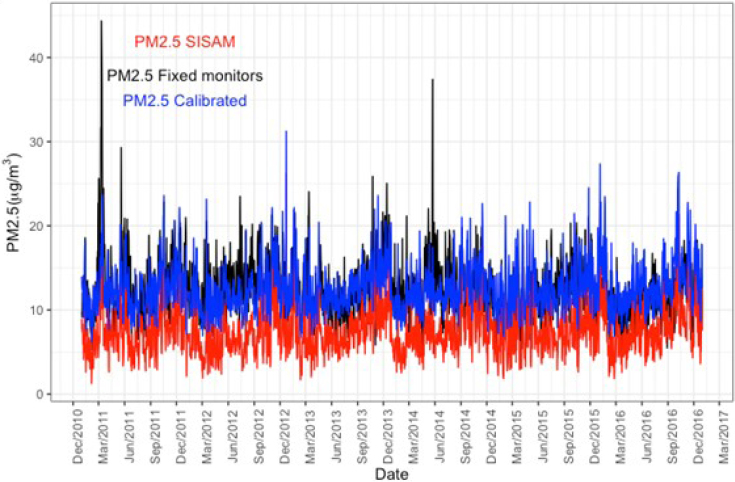
Time series of daily data on particulate matter with a diameter of less than 2.5 μm (PM2.5) from the Environmental Information System Integrated with Environmental Health (SISAM) (original and calibrated) measured by monitoring stations (PM2.5 Fixed monitors). Salvador (BA), 2011 to 2016.

In Figures 3 and 4 we demonstrate the comparison of the daily time series between the modeled SISAM data and the mean concentrations of the measured PM2.5 data. SISAM modeled data underestimated PM2.5 levels of Salvador during the study period compared with the mean measurements from Cetrel monitoring stations, with a statistically significant daily mean difference of 5.021 μg/m^3^ (p<0.001) (Figure 4). This result indicates a systematic bias. Despite this underestimation, both data showed a similar trend and seasonality pattern. The concordance correlation coefficient was low, 0.20 (95%CI 0.18–0.22), suggesting little agreement between the two data sources. During atypical pollution events, when PM2.5 concentrations were above 30 μg/m^3^, the discrepancy between SISAM measurements and means of Cetrel monitors increased. The relative difference between the maximum values of the time series was approximately 50%. For the cross-correlation between the two time series, the correlation coefficient at lag 0 was 0.45, decreasing as the lag increased.

Fixed monitors: refers to PM2.5 data measured by monitoring stations; SISAM: refers to SISAM estimated data.

*The mean difference between the SISAM data and the fixed monitors was 5.021 and the root mean square error (RMSE) was 6.1; the correction consisted of adding this factor (5.021) to the SISAM data. The calibrated PM2.5 time series is in blue, with RMSE of 3.42.

In Figure 3A, a positive relationship between the variables is observed. Overall, when PM2.5 concentrations measured by monitoring stations increase, SISAM estimates also increase. According to the analysis using the Bland-Altman plot (Figure 3B), we verified that, although most data points were within the limits of agreement, a statistically significant negative correlation (p=-0.23, p<0.001) between the differences (Y) and the means (X) of the PM2.5 measurements indicates heteroscedasticity of the errors. This bias was more pronounced for mean values above 20 μg/m^3^, highlighting the need for calibration to increase the accuracy of the estimates.

This bias was used as a correction factor for the SISAM measurements. When adding 5.021 μg/m^3^, the calibrated series showed better alignment with the measured data, maintaining the variability of the original SISAM data (Figure 4).

**Figure 5:**
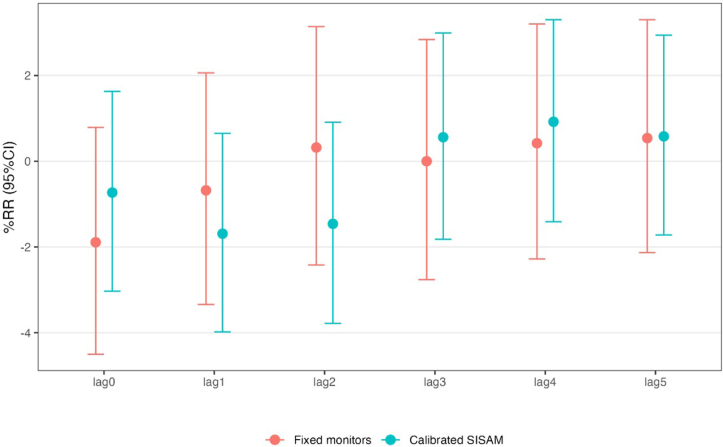
Percentage change in risk (95% confidence interval) of deaths associated with a 10 μg/m^3^ increase in levels of particulate matter with a diameter of less than 2.5 μm — PM2.5 (Fixed monitors and Environmental Information System Integrated with Environmental Health — SISAM). Salvador (BA), 2011 to 2016.

Although the results indicated a systematic bias for the SISAM data and errors associated with the magnitude of the mean PM2.5 estimate, when comparing the health effects, we observed no statistically significant differences between the risk estimates based on the PM2.5 means from the Cetrel fixed monitors and the calibrated PM2.5 data (Figure 5). For each 10 μg/m^3^ increase in PM2.5, the estimated risk of death was 0.5% (95%CI −2.1–3.3%) associated with five-day lagged exposure.

*%RR: (relative risk −1)*100

**Figure 6:**
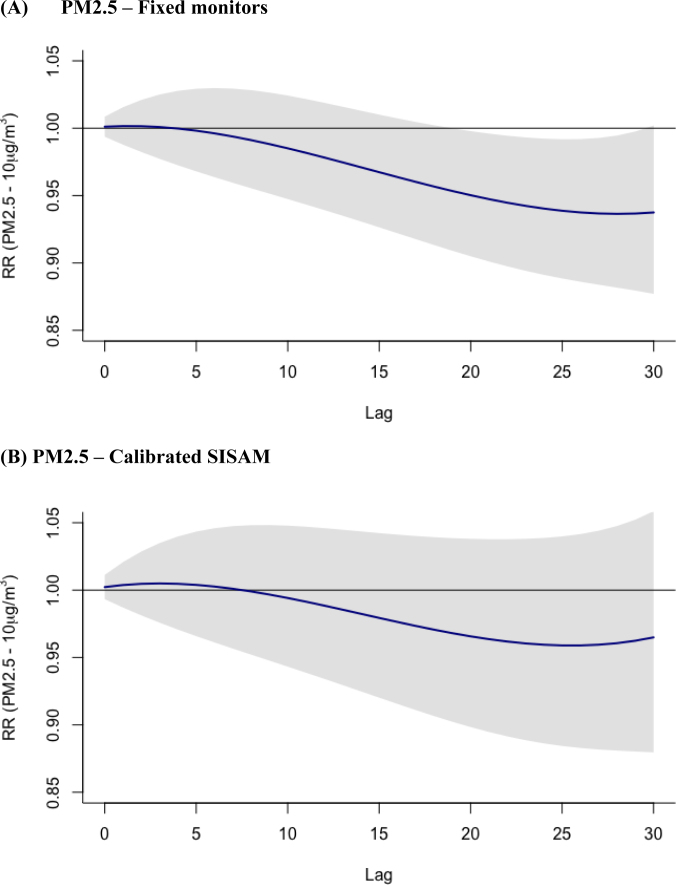
Cumulative relative risk (RR) (95% confidence interval) of deaths associated with a 10 μg/m^3^ increase in the levels of particulate matter with a diameter of less than 2.5 μm — PM2.5 (Fixed monitors, Environmental Information System Integrated with Environmental Health — Calibrated SISAM). Salvador (BA), 2011 to 2016.

The exposure-lag-response curves for the cumulative effects of PM2.5 over 30 days indicated a low risk of death in the first few days after exposure, with greater uncertainty in the results derived from calibrated SISAM data compared with data from the fixed monitors (Figure 6). The concordance coefficient between the RR estimates of the measured data and the calibrated data from SISAM was 0.80 (95%CI 0.70–0.88), indicating good agreement between the risk measurements.

Cumulative lag structures from lag 0 to lag 30, with restriction of a second-degree polynomial. The shadow area represents the 95%CI.

As most of the estimated RRs were not statistically significant, we cannot state that exposure to PM2.5 is a risk factor for mortality in Salvador during the studied period.

## DISCUSSION

According to our results, we found a significant underestimation of PM2.5 levels modeled by remote sensing and the need for calibration to ensure data accuracy in health risk assessment studies. Despite the identified systematic bias and heteroscedastic errors, the SISAM PM2.5 estimates were effectively adjusted by the proposed simple calibration method, demonstrating the potential of remotely sensed modeled data to provide valuable information on air pollution exposure and its health implications. The critical situation of air pollution in Salvador stands out, with PM2.5 levels exceeding WHO guidelines^39^.

Although the agreement between the mean data from fixed monitors and SISAM was low, we observed high agreement regarding the effects estimated by the regression models, and the differences between the relative risk estimates were not statistically significant. These results suggest that data from SISAM can be a reliable source for assessing the effects of air pollution on health, especially considering that the calibration of the time series did not alter the trend, seasonality, and variability of the series.

The application of modeled data to assess air pollution exposure for health studies is a growing field within epidemiology^40–42^. However, using modeled air quality data without proper and site-specific validation may lead to underestimations or overestimations of the health impacts of air pollution. There are different calibration methods for modeled data, such as the one proposed in this study, which effectively corrected the distribution of the modeled SISAM data^43,44^.

Although most of the estimated risks were not significant, daily exposure to low concentrations of pollutants represents long-term accumulated exposure that can impact quality of life^45–48^. Time series epidemiological studies have been used to quantify the health risks associated with short-term exposure to air pollutants^13–17^. Authors of a study conducted in São Paulo found that PM10, NO_2_, and CO pollutants were significantly associated with non-accidental and cause-specific deaths in both single lag and cumulative lag models^16^. For non-accidental mortality, in Salvador, the estimated risk associated with exposure to PM2.5 was 0.5% (95%CI −2.1–3.3) and, in the city of São Paulo, it was 0.6% (95%CI 0.4–0.8%).

The SISAM platform should be highlighted, because it operates according to open data principles. These data contribute to air quality monitoring, risk communication, and research into the impacts of air pollution on health outcomes. Risk communication in Brazil is based on Resolution No. 491 of the National Environment Council (*Conselho Nacional do Meio Ambiente* – CONAMA), which uses as a reference the air quality guide values recommended by the WHO in 2005, which indicates the standard for daily exposure to PM2.5 as 25 μg/m^3^. This concentration was reviewed and updated by WHO in 2021 to the daily exposure value of 15 μg/m^3^. For the period studied in Salvador, the mean corrected PM2.5 value was 12.5 μg/m^3^ and the maximum value was 31.2 μg/m^3^. The maximum value exceeded the WHO limit twice, which indicates a state of alert for certain neighborhoods in Salvador, considering vulnerable groups such as pregnant women, children under five years of age, and people with comorbidities.

It is worth highlighting that the case study of the municipality of Salvador is a local application, characterized by stable meteorological conditions and low variability of PM2.5 concentrations, factors that contribute to better results of satellite-modeled data for air quality assessments in the region.

Although this satellite-modeled data tool has been used in the United States of America since 2009 in environmental health studies^23^, some gaps should be discussed, such as:

1. Ground-level particulate matter concentrations are specific measurements at fixed locations, which may be more representative of the breathing zone compared to the raw aerosol optical depth (AOD) value, which represents the integration of the entire atmospheric column;
2. Ground-level particulate matter concentrations are continuously monitored (e.g., hourly, every three hours over a 24-hour period), whereas AOD is measured as the satellite passes, which is typically once a day for the most frequently used instruments; therefore, the satellite-derived measurement does not necessarily represent the variability at each location;
3. For health studies, it is important to know the composition of the particulate matter, as this helps understanding the toxicity to human health, and the modeled datum does not allow this knowledge;
4. Satellite data is underrepresented on days with high cloud cover, as this masks data retrieval abilities.

In this sense, Salvador is a privileged city, because it presents low variability in meteorological parameters that can interfere with AOD measurements and their modeling. Data from the SISAM platform for the municipality of Salvador are valid for estimating the health risks of exposure to PM2.5 through time series models, although they underestimate the mean daily concentration of the pollutant. Thus, in this study we highlight the underestimation of PM2.5 levels by SISAM and recommend the application of a correction factor of 5 μg/m^3^ to improve accuracy. Although there are uncertainties inherent in modeled data, as is the case with SISAM, it is worth noting that the platform is still one of the best for air quality studies when continuous monitoring with advanced sampling techniques is not possible.

## References

[1] World Health Organization Ambient (outdoor) air pollution [Internet].

[2] Pope CA, Dockery DW (2006). Health effects of fine particulate air pollution: lines that connect. J Air Waste Manag Assoc.

[3] Samet J, Krewski D (2007). Health effects associated with exposure to ambient air pollution. J Toxicol Environ Health A.

[4] Zhang Y, Tingting Y, Huang W, Yu P, Chen G, Xu R (2024). Health impacts of wildfire smoke on children and adolescents: a systematic review and meta-analysis. Curr Environ Health Rep.

[5] Organização Pan-Americana da Saúde (2018). Não polua o meu futuro! O impacto do ambiente na saúde das crianças [Internet].

[6] Quintanilha WFL, Maia ML, Bertoncini BV, Ribeio JP, Cassiano DR, Sousa FW (2021). Evaluation of atmospheric NO2 levels in public transport corridors. Transportes.

[7] Lim SS, Vos T, Flaxman AD, Danaei G, Shibuya K, Adair-Rohani H (2012). A comparative risk assessment of burden of disease and injury attributable to 67 risk factors and risk factor clusters in 21 regions, 1990–2010: a systematic analysis for the Global Burden of Disease Study 2010. Lancet.

[8] Feng S, Gao D, Liao F, Zhou F, Wang X (2016). The health effects of ambient PM2.5 and potential mechanisms. Ecotoxicol Environ Saf.

[9] United States. Environmental Protection Agency (2002). Third external review draft of air quality criteria for particulate matter.

[10] Cohen AJ, Anderson R, Ostro B, Pandey KD, Krzyzanowski M, Künzli N, Ezzati M, Lopez AD, Rodgers A, Murray CJL (2004). Comparative quantification of health risks. Global and regional burden of disease attributable to selected major risk factors.

[11] Souza PA, Mello WZ, Mariani RL, Sella SM (2010). Caracterização do material particulado fino e grosso e composição da fração inorgânica solúvel em água em São José dos Campos (SP). Quím Nova.

[12] Jacobson LSV, Hacon SS, Castro HA, Ignotti E, Artaxo P, Leon ACMP (2012). Association between fine particulate matter and the peak expiratory flow of schoolchildren in the Brazilian subequatorial Amazon: a panel study. Environ Res.

[13] Ignotti E, Hacon SS, Junger WL, Mourão D, Longo K, Freitas S (2010). Air pollution and hospital admissions for respiratory diseases in the subequatorial Amazon: a time series approach. Cad Saude Publica.

[14] Romieu I, Gouveia N, Cifuentes LA, Leon AP, Junger W, Vera J (2012). Multicity study of air pollution and mortality in Latin America (the ESCALA study). Res Rep Health Eff Inst.

[15] Atkinson RW, Kang S, Anderson HR, Mills IC, Walton HA (2014). Epidemiological time series studies of PM2.5 and daily mortality and hospital admissions: a systematic review and meta-analysis. Thorax.

[16] Costa AF, Hoek G, Brunekreef B, Leon ACMP (2017). Air pollution and deaths among elderly residents of São Paulo, Brazil: an analysis of mortality displacement. Environ Health Perspect.

[17] Qu Y, Pan Y, Niu H, He Y, Li M, Li L (2018). Short-term effects of fine particulate matter on non-accidental and circulatory diseases mortality: a time series study among the elder in Changchun. PLoS One.

[18] Liu C, Cai J, Chen R, Sera F, Guo Y, Tong S (2022). Coarse particulate air pollution and daily mortality: a global study in 205 cities. Am J Respir Crit Care Med.

[19] Vormittag EMPAA, Cirqueira SSR, Wicher H, Saldiva PHN (2021). Análise do monitoramento da qualidade do ar no Brasil. Estud Av.

[20] Dominici F, Sheppard L, Clyde M (2003). Health effects of air pollution: a statistical review. Int Stat Rev.

[21] Lepot M, Aubin JB, Clemens FHLR (2017). Interpolation in time series: an introductive overview of existing methods, their performance criteria and uncertainty assessment. Water.

[22] Schneider R, Masselot P, Vicedo-Cabrera AM, Sera F, Blangiardo M, Forlani C (2022). Differential impact of government lockdown policies on reducing air pollution levels and related mortality in Europe. Sci Rep.

[23] Sorek-Hamer M, Just AC, Kloog I (2016). Satellite remote sensing in epidemiological studies. Curr Opin Pediatr.

[24] Brasil (2010). Instituto Brasileiro de Geografia e Estatística. Atlas do censo demográfico 2010 [Internet].

[25] Ostro B (2004). Outdoor air pollution: assessing the environmental burden of disease at national and local levels.

[26] Instituto Nacional de Pesquisas Espaciais (2023). sistema de transferência de dados [Internet].

[27] Christophe Y, Bennouna Y, Schulz M, Eskes HJ, Basart S, Benedictow A (2019). Validation report of the CAMS global reanalysis of aerosols and reactive gases, years 2003–2018.

[28] Eskes H, Huijnen V, Arola A, Benedictow A, Blechschmidt AM, Botek E (2015). Validation of reactive gases and aerosols in the MACC global analysis and forecast system. Geosci Model Dev.

[29] Inness A, Ades M, Agustí-Panareda A, Barré J, Benedictow A, Blechschmidt AM (2019). The CAMS reanalysis of atmospheric composition. Atmos Chem Phys.

[30] Wang Y, Ma YF, Eskes H, Inness A, Flemming J, Brasseur GP (2020). Evaluation of the CAMS global atmospheric trace gas reanalysis 2003–2016 using aircraft campaign observations. Atmos Chem Phys.

[31] Bland JM, Altman DG (1986). Statistical methods for assessing agreement between two methods of clinical measurement. Lancet.

[32] Lin LI (1989). A concordance correlation coefficient to evaluate reproducibility. Biometrics.

[33] Nickerson CAE (1997). A note on "A concordance correlation coefficient to evaluate reproducibility.". Biometrics.

[34] R Core Team (2022). R: A language and environment for statistical computing [Internet].

[35] Stevenson M, Sergeant E, Heuer C, Nunes T, Marshall J, Sanchez J Package epiR: tools for the analysis of epidemiological data [Internet].

[36] Datta D (2017). blandr: a Bland-Altman MethodComparison package for R [Internet].

[37] Wood SN (2017). Generalized additive models: an introduction with R.

[38] Gasparrini A, Armstrong B, Kenward MG (2010). Distributed lag non-linear models. Stat Med.

[39] World Health Organization (2021). WHO global air quality guidelines: particulate matter (PM2.5 and PM10), ozone, nitrogen dioxide, sulfur dioxide and carbon monoxide [Internet].

[40] Hu Z (2009). Spatial analysis of MODIS aerosol optical depth, PM2.5, and chronic coronary heart disease. Int J Health Geogr.

[41] Kloog I, Zanobetti A, Nordio F, Coull BA, Baccarelli AA, Schwartz J (2015). Effects of airborne fine particles (PM2.5) on deep vein thrombosis admissions in northeastern United States. J Thromb Haemost.

[42] Kloog I, Melly SJ, Ridgway WL, Coull BA, Schwartz J (2012). Using new satellite based exposure methods to study the association between pregnancy pm2.5 exposure, premature birth and birth weight in Massachusetts. Environ Health.

[43] Teutschbein C, Seibert J (2012). Bias correction of regional climate model simulations for hydrological climate-change impact studies: review and evaluation of different methods. J Hydrol.

[44] Hempel S, Frieler K, Warszawski L, Schewe J, Piontek F (2013). A trend-preserving bias correction – the ISI-MIP approach. Earth Syst Dynam.

[45] Pope CA, Burnett RT, Thun MJ, Calle EE, Krewski D, Ito K (2002). Lung cancer, cardiopulmonary mortality, and long-term exposure to fine particulate air pollution. JAMA.

[46] Cohen AJ, Brauer M, Burnett R, Anderson HR, Frostad J, Estep K Estimates and 25-year trends of the global burden of disease attributable to ambient air pollution: an analysis of data from the Global Burden of Diseases Study 2015. Lancet.

[47] Analitis A, De’ Donato F, Scortichini M, Lanki T, Basagana X, Ballester F (2018). Synergistic effects of ambient temperature and air pollution on health in Europe: results from the PHASE project. Int J Environ Res Public Health.

[48] Abdillah SF, Wang YF (2023). Ambient ultrafine particle (PM0.1): sources, characteristics, measurements and exposure implications on human health. Environ Res.

