# Supplementary material for: Performance of fine particulate matter data on air quality in an epidemiological study in Salvador, Brazil
Source: Rev Bras Epidemiol. 2024 Dec 16;27:e240068. doi: 10.1590/1980-549720240068 (PMC11654642; doi:10.1590/1980-549720240068)
Supplement: Translation

# Translation: Desempenho dos dados de material particulado fino sobre a qualidade do ar em estudo epidemiológico em Salvador, Brasil

**DOI:** 10.1590/1980-549720240068.2

**Authors:** Ludmilla Viana Jacobson, Sandra Hacon, Vanúcia Schumacher, Clarcson Plácido Conceição Dos Santos, Nelzair Vianna

**Affiliations:** I Universidade Federal Fluminense, Departamento de Estatística – Niterói (RJ), Brasil.; II Fundação Oswaldo Cruz, Escola Nacional de Saúde Pública Sérgio Arouca – Rio de Janeiro (RJ), Brasil.; III Instituto Nacional de Pesquisas Espaciais, Centro de Previsão do Tempo e Estudos Climáticos, São José dos Campos (SP), Brasil.; IV Escola Bahiana de Medicina e Saúde Pública, Programa de Pós-Graduação em Medicina e Saúde Humana – Salvador (BA), Brasil.; V Fundação Oswaldo Cruz, Laboratório de Biologia Molecular e Bioestatística – Salvador (BA), Brasil.

**Keywords:** Poluição do ar, Saúde pública, Séries temporais, Sistemas de informações

## Abstract

**Objetivo:**

Avaliar o desempenho das concentrações de material particulado com diâmetro inferior a 2,5 μm (PM2,5) derivadas de satélite em comparação com medições de estações de monitoramento no município de Salvador, bem como as implicações dessas estimativas para as associações de PM2,5 com a mortalidade diária não acidental.

**Métodos:**

Trata-se de estudo de séries temporais diária que cobre o período de 2011 a 2016. Foi proposto um fator de correção para melhorar a acurácia entre as duas fontes de dados. Os efeitos do PM2,5 foram estimados em modelos aditivos generalizados de Poisson, combinados com uma abordagem de defasagem distribuída.

**Resultados:**

Os resultados sugerem que os dados derivados de satélite subestimaram os níveis de PM2,5 em comparação com as medições médias terrestres. No entanto, a aplicação de um fator de correção melhorou a acurácia entre os dados. Os riscos relativos estimados com base nos dados derivados de satélite não apresentaram diferenças significativas quando comparados com aqueles baseados nas médias dos monitores.

**Conclusão:**

O estudo destaca a importância de validar dados de PM2,5 modelados por satélite para avaliar e compreender os impactos na saúde. O desenvolvimento de modelos que utilizam sensoriamento remoto para estimar PM2,5 permite a quantificação dos riscos à saúde decorrentes da exposição.

## INTRODUÇÃO

A poluição do ar é uma ameaça ambiental com múltiplos impactos para a saúde humana. A Organização Mundial da Saúde (OMS) chama a atenção para a carga de doença associada à poluição atmosférica. Milhões de mortes prematuras por acidente vascular cerebral, doenças cardíacas, câncer de pulmão e doenças respiratórias crônicas e agudas, incluindo asma, poderiam ser evitadas ao se reduzirem os níveis de poluição do ar^1^. Populações vulneráveis, incluindo as crianças, idosos, gestantes e pessoas com doenças crônicas, estão em maior risco^2–4^. No Brasil, a Organização Pan-Americana da Saúde reportou 51 mil mortes anuais associadas à poluição atmosférica^5,6^.

A exposição ao material particulado com diâmetro inferior a 2,5 μm (PM2,5) foi investigada em muitos estudos e tem se mostrado um indicador robusto de risco à saúde, associado a diferentes fontes de emissão^7,8^. Vale ressaltar que o PM2,5 compreende o material inalável PM10. Em áreas urbanas de países desenvolvidos, a fração de PM2,5 para PM10 variou de 0,5 a 0,8^9^. No estudo realizado por Cohen et al.^10^ em diversas áreas urbanas do mundo, foi adotada a fração de 0,5xPM10 para estimar o PM2,5 e calcular o número de mortes prematuras associadas à exposição ao poluente. No Brasil, em estudo realizado em área urbana, a média da razão PM2,5/PM10 foi de 0,52^11^. Tanto o PM2,5 quanto o PM_10_ podem ser inalados, porém o PM2,5 tem maior potencial de risco à saúde, pois as partículas finas podem penetrar profundamente no trato respiratório e atingir os pulmões^12^.

Os estudos epidemiológicos de séries temporais têm sido utilizados para quantificar os riscos associados à exposição a poluentes atmosféricos e desfechos de saúde^13–18^. Um dos relevantes desafios na investigação dos efeitos da poluição do ar sobre a saúde da população nas principais capitais do Brasil é a falta de monitoramento contínuo de poluentes atmosféricos. No Brasil, a rede de monitoramento da qualidade do ar é restrita, o que representa uma limitação para o desenvolvimento de estudos epidemiológicos. Dos 26 estados brasileiros, 11 possuem sistema de monitoramento da qualidade do ar, dos quais 80% estão localizados na Região Sudeste, mas apresentam informações incompletas e acesso limitado referente à qualidade do ar^19^. Para estudos epidemiológicos de séries temporais, o período do estudo e a disponibilidade de dados de qualidade do ar é um fator importante para a redução das incertezas, embora indicadores *proxy* e informações de modelos preditivos também possam ser usados desde que sejam discutidas as suas limitações^20–22^.

O Instituto Nacional de Pesquisas Espaciais (Inpe), em colaboração com outras instituições brasileiras, desenvolveu o Sistema de Informações Ambientais Integrado à Saúde Ambiental (SISAM) com o objetivo de auxiliar programas de saúde sobre os impactos das emissões de poluentes atmosféricos na saúde humana. O SISAM é uma plataforma *on-line* que disponibiliza as concentrações dos poluentes atmosféricos, estimadas por sensoriamento remoto com resolução espacial de aproximadamente 12,5 km, que permite uma caracterização espaçotemporal da variabilidade da exposição diária para todos os municípios do Brasil, sem custo para o usuário^23^.

O objetivo deste trabalho foi avaliar o desempenho das estimativas de PM2,5 provenientes da plataforma SISAM em comparação com medições de estações de monitoramento no município de Salvador no estado da Bahia, bem como suas implicações para as associações de PM2,5 com a mortalidade não acidental.

## MÉTODOS

### Desenho e área de estudo

Estudo ecológico de séries temporais diárias, no período de 2011 a 2016.

A pesquisa foi realizada em Salvador, segunda maior cidade do nordeste do Brasil e a quinta mais populosa do país, com população de aproximadamente 2,42 milhões de acordo com o Censo de 2022^24^. O município é dividido em dez prefeituras-bairros e estas subdivididas em 163 bairros.

No período de 2011 a 2016, o monitoramento da qualidade do ar em Salvador foi gerenciado pela Cetrel, empresa privada brasileira especializada em monitoramento ambiental. Nesse período, a rede de monitoramento da qualidade do ar de Salvador era composta de oito estações de monitoramento fixas, estrategicamente localizadas em toda a cidade. Essas estações forneciam dados com resolução temporal de uma hora sobre níveis de poluentes como PM10, SO_2_, CO, O_3_ e NO_2_; além de parâmetros meteorológicos, como velocidade e direção do vento, temperatura, umidade e precipitação. Dentre as estações de monitoramento, cinco estavam em operação a partir de 2011, a saber: Campo Grande, Dique do Tororó, CAB/Dique do Paralela, Pirajá, Rio Vermelho. As três estações restantes — como as estações Av. ACM/Detran, Av. Barros Reis e Itaigara — passaram a funcionar em 2013. Até o início deste estudo, as medições diárias de poluentes atmosféricos disponíveis em Salvador eram somente do período de 2011 a 2016. A área de estudo e a localização específica das estações de monitoramento estão apresentadas na Figura 1.

**Figura 1:** Localização das estações de monitoramento fixas de Salvador (BA), 2011 a 2016.

PIR: Pirajá; PAR: estação CAB/Paralela; CAM: Campo Grande; RIO: Rio Vermelho; DIQ: Dique do Tororo; ITA: Itaigara; BAR: Barros Reis; ACM: Av. ACM/Detran.

### Bases de dados

#### Dados medidos de poluentes atmosféricos e parâmetros meteorológicos

Os dados de PM10, temperatura e umidade foram obtidos das estações de monitoramento da Cetrel para o período de 2011 a 2016. Diante da ausência de medições diretas de PM2,5, foi utilizada a proporção de 0,5 para PM2,5/PM10, comumente aceita para áreas urbanas e conforme recomendado por Ostro^25^. Essa abordagem permitiu estimar as concentrações de PM2,5 com base nos níveis de PM_10_ registrados.

#### Plataforma do Sistema de Informações Ambientais Integrado à Saúde Ambiental (SISAM)

A plataforma SISAM apresenta planilhas com dados meteorológicos e qualidade do ar para todos os estados e municípios, por data e hora^26^. O SISAM tem como fonte o Copernicus Atmosphere Monitoring Service (CAMS), conjunto de dados de reanálise global da composição atmosférica produzido pelo European Centre for Medium-Range Weather Forecasts com o Integrated Forecasting System atualizado. O CAMS combina informações de observações *in situ* e de satélite com modelos computacionais da atmosfera para gerar uma estimativa, a mais precisa possível, de gases e aerossóis atmosféricos. A validação do CAMS é realizada periodicamente e coordenada pela Royal Dutch Meteorological Organization^27,28^. Innes et al.^29^ avaliaram o desempenho das reanálises do CAMS comparando versões anteriores, e Wang et al.^30^ validaram o CAMS usando medições de aeronaves em diferentes partes do mundo, incluindo o Brasil.

Para as análises deste estudo e caracterização espaçotemporal da variabilidade da exposição diária em Salvador, foi considerada a média diária de PM_2,5_ no período de 2011 a 2016.

#### Dados de óbitos

O desfecho de saúde investigado foi o número total de óbitos diários por todas as causas não acidentais. Os dados de óbito foram aqueles disponibilizados no Sistema de Informação sobre Mortalidade por meio do Departamento de Informática do Sistema Único de Saúde. Foram excluídas das análises as causas acidentais, aquelas registradas na declaração de óbitos como causa básica classificada segundo os códigos S00 a T98, V01 a Y98, Z00 a Z99, U00 a U99 (CID10).

### Análise dos dados

#### Medidas de concordância

A concordância entre os dados diários de PM2,5 modelados do SISAM e os dados medidos das estações de monitoramento da Cetrel foi investigada por meio do teste *t* para amostras pareadas, do método de Bland-Altman^31^ e do coeficiente de correlação de concordância^32,33^. O teste *t* pareado foi utilizado para avaliar diferenças entre os métodos de medição quanto ao viés sistemático. O método Bland-Altman consiste no gráfico de dispersão entre as diferenças (Diferença=PM2,5 SISAM – PM2,5 Medidos) e as médias [Média=(PM2,5 SISAM–PM2,5 Medido)/2]. Nesse método, as médias são usadas como estimativas do valor verdadeiro do PM2,5 e, portanto, é possível investigar a relação dos erros com o valor verdadeiro de PM2,5. O método também apresenta os limites de concordância calculado por meio da média das diferenças (viés) e seu desvio padrão (sd), como viés±1,96sd. A heterocedasticidade do erro foi avaliada pelo coeficiente de correlação de Pearson entre as diferenças (Y) e as médias (X). O coeficiente de correlação de concordância (CCC) foi utilizado para avaliar o quanto os métodos diferem entre si.

As análises foram realizadas no programa R 3.4.2.2^34^, bibliotecas *epiR* ^35^ e *blandr* ^36^. Foi adotado nível de significância de 5% na interpretação dos resultados dos testes de hipóteses.

#### Calibração dos dados do Sistema de Informações Ambientais Integrado à Saúde Ambiental (SISAM)

As séries temporais diárias de PM2,5 observado (dados da Cetrel) e modelado (dados SISAM) foram comparadas por meio de gráficos e estatísticas descritivas. Em seguida, o indicador diferença absoluta (Δ_*t*_ =PM2,5observado_t_ – PM2,5modelado_t_) foi calculado.

Na calibração dos dados, foi proposta a definição de um termo aditivo baseado na média do indicador diferença absoluta (Δ_*t*_), no período de 2011 a 2016. O PM2,5 calibrado foi calculado pela equação:


$$PM_{2,5}calibra{\text{do}}_{t}=PM_{2,5t}+\bar{x}$$

Em que:

$\bar{x}=\frac{\sum_{t=1}^{n}\Delta_{t}}{n}$ , PM_2,5t_ é o PM_2,5_ modelado do SISAM no dia t; e "n" é o total de dias no período de 2011 a 2016.

Este método foi utilizado para corrigir o viés observado nos dados do SISAM.

#### Análise estatística

Para avaliar a *performance* dos dados do SISAM em estudos epidemiológicos em Salvador, foi proposto um estudo ecológico de séries temporais diárias no período de 2011 a 2016. Foram comparados os efeitos na saúde baseados nos dados medidos pelos monitores fixos com os efeitos estimados por meio dos dados do SISAM calibrados.

Na análise de séries temporais foram ajustados modelos aditivos generalizados (MAG), considerando-se a distribuição de Poisson com função de ligação logarítmica, combinada com o modelo de defasagem distribuída^16,37,38^. A variável resposta foi a contagem diária do número de óbitos, e as variáveis explicativas foram a indicadora do dia calendário para o ajuste da tendência e sazonalidade, temperatura, umidade, indicadora do dia da semana, indicadora de feriados nacionais e PM2,5.

A tendência temporal e sazonalidade foram ajustadas no modelo por uma *thin plate spline*, incluída na regressão com 4 graus de liberdade (gl) por ano; a temperatura média diária com defasagem de três dias (Lag 3) foi ajustada por uma *thin plate spline* com 2 gl; a umidade média diária de Lag 3 foi ajustada por uma *thin plate spline* com 2 gl. Diversos ajustes foram avaliados para a tendência temporal e sazonalidade, variando o tipo da *spline* e graus de liberdade (gl: 4 a 6 por ano). Da mesma forma para a temperatura e umidade, em que além das médias diárias foram avaliadas as defasagens de Lag 1, Lag 2 e Lag 3. A escolha do número de gl e do melhor ajuste para o modelo foi baseada no critério de informação de Akaike (AIC), em gráficos de dispersão dos resíduos de *deviance* e periodograma. Após a escolha do melhor ajuste para a tendência e sazonalidade do tempo, temperatura e umidade, foram incluídas as variáveis indicadoras de dia da semana e feriado nacional.

Como os efeitos da poluição do ar sobre a mortalidade podem ocorrer no mesmo dia da exposição ou em dias posteriores, o PM2,5 foi ajustado por uma função polinomial de defasagem distribuída. O ajuste considerou um polinômio de segundo grau e defasagem de até 30 dias (lag 0-30). Além disso, foram estimados também os efeitos *Single Lag* de zero a cinco dias. Os resultados foram apresentados como risco relativo (RR) e incremento percentual do risco de óbito para cada aumento de 10 μg/m^3^ de PM2,5, assim como os respectivos intervalos de confiança de 95% (IC95%). Ademais, foi apresentada a curva exposição-lag-resposta dos efeitos do PM2,5 sobre a mortalidade acumulados em 30 dias de exposição.

## RESULTADOS

Durante o período do estudo, a concentração média de PM2,5, medida pelos monitores fixos da Cetrel e calculada com base nos dados medidos de PM10, foi de 12,5 μg/m^3^, enquanto os dados de PM2,5 modelados do SISAM foram, em média, de 7,5 μg/m^3^±2,9 μg/m^3^. As médias anuais de PM2,5, apresentadas na Figura 2A, ultrapassaram a recomendação da OMS para a média anual de qualidade do ar de 5 μg/m^3 39^. Foram registradas, em média, 35±6,1 mortes não acidentais por dia. Além disso, a temperatura média diária ficou em torno de 26°C, com umidade relativa de 73% (Tabela 1). As variáveis ambientais apresentaram baixas correlações com as mortes não acidentais (Figura 2B).

**Figura 2:** Médias anuais dos dados de material particulado com diâmetro inferior a 2,5 μm (PM2,5) do Sistema de Informações Ambientais Integrado à Saúde Ambiental (SISAM) e das estações de monitoramento (A) e correlações com as variáveis meteorológicas (B). Salvador (BA), 2011 a 2016.

**Tabela 1:** Estatísticas resumo dos dados diários das variáveis de exposição, meteorológicas e de saúde. Salvador, 2011 – 2016.

	Medidas Diárias							
	Min	P25	P50	Méd	P75	Max	var	sd
PM2,5 (μg/m^3^) – SISAM	1,3	5,6	7,1	7,5	9,0	26,2	8,4	2,9
PM2,5 (μg/m^3^) – Monitores fixos	4,8	10,1	12,2	12,5	14,5	44,4	12,7	3,6
Temperatura (°C)	22,0	25,3	26,4	26,3	27,4	29,3	1,9	1,4
Umidade (%)	59,1	69,1	72,2	73,0	76,3	93,5	31,2	5,6
Mortes por causas não acidentais	17,0	31,0	35,0	35,1	39,0	68,0	37,5	6,1

Min: valor mínimo; Max: valor máximo; P25: percentil 25; P50: percentil 50; Méd: média; P75: percentil 75; var: variância; sd: desvio padrão.

As Figuras 3 e 4 mostram a comparação das séries temporais diárias entre os dados modelados do SISAM e as concentrações médias dos dados medidos de PM2,5. Os dados modelados do SISAM subestimaram os níveis de PM2,5 de Salvador no período de estudo em comparação com as medições médias das estações de monitoramento da Cetrel, com diferença média diária estatisticamente significativa de 5,021 μg/m^3^ (p<0,001) (Figura 4). Esse resultado indica um viés sistemático. Apesar dessa subestimação, ambos os dados exibiram uma tendência e um padrão de sazonalidade semelhantes. O coeficiente de correlação de concordância foi baixo, de 0,20 (IC95% 0,18–0,22), sugerindo uma pequena concordância entre as duas fontes de dados. Durante eventos atípicos de poluição, quando as concentrações de PM2,5 estiveram acima de 30 μg/m^3^, a discrepância entre as medições do SISAM e das médias dos monitores da Cetrel aumentou. A diferença relativa entre os valores máximos das séries temporais foi de aproximadamente 50%. Para a correlação cruzada entre as duas séries temporais, o coeficiente de correlação na defasagem 0 foi de 0,45, diminuindo à medida que a defasagem aumentou.

**Figura 3:** Comparação entre os dados modelados de material particulado com diâmetro inferior a 2,5 μm (PM2,5) (Sistema de Informações Ambientais Integrado à Saúde Ambiental — SISAM) e os dados medidos pelas estações de monitoramento (Monitores Fixos). Salvador (BA), 2011 a 2016.

Monitores Fixos: refere-se aos dados de PM2,5 medidos pelas estações de monitoramento; SISAM: refere-se aos dados estimados SISAM.

**Figura 4:** Séries temporais dos dados diários de material particulado com diâmetro inferior a 2,5 μm (PM_2,5_) do Sistema de Informações Ambientais Integrado à Saúde Ambiental (SISAM) (Original e Calibrado) medidos pelas estações de monitoramento (PM2,5 Monitores Fixos). Salvador (BA), 2011 a 2016.

*A diferença média entre os dados do SISAM e dos monitores fixos foi de 5,021 e o erro quadrático médio (RSME) foi de 6,1; a correção consistiu em somar este fator (5,021) aos dados do SISAM. Em azul está a série temporal de PM2,5 calibrado, com RMSE de 3,42.

Na Figura 3A, observa-se relação positiva entre as variáveis. De maneira geral, quando as concentrações de PM_2,5_ medidas pelas estações de monitoramento aumentam, as estimativas do SISAM também aumentam. A análise por meio do gráfico de Bland-Altman (Figura 3B) demonstrou que, embora a maioria dos pontos dos dados estivesse dentro dos limites de concordância, uma correlação negativa estatisticamente significativa (p=-0,23, p<0,001) entre as diferenças (Y) e as médias (X) das medidas de PM2,5 indica heterocedasticidade dos erros. Esse viés foi mais pronunciado para valores médios acima de 20 μg/m^3^, ressaltando a necessidade de calibração para aumentar a acurácia das estimativas.

O viés acima identificado foi utilizado como fator de correção para as medidas do SISAM. Ao adicionar 5,021 μg/m^3^, a série calibrada apresentou melhor alinhamento com os dados medidos, mantendo a variabilidade dos dados originais do SISAM (Figura 4).

Embora os resultados tenham indicado um viés sistemático para os dados do SISAM e erros associados à magnitude da estimativa de média do PM2,5, ao se compararem os efeitos na saúde não foram observadas diferenças estatisticamente significativas entre as estimativas de risco baseadas nas médias de PM2,5 dos monitores fixos da Cetrel e os dados calibrados de PM2,5 (Figura 5). Para cada aumento de 10 μg/m^3^ no PM2,5, o risco estimado de óbito foi de 0,5% (IC95% −2,1–3,3%) associado à exposição defasada em cinco dias.

**Figura 5:** Variação percentual do risco (intervalo de confiança de 95%) de óbitos associado ao aumento 10 μg/m^3^ nos níveis de material particulado com diâmetro inferior a 2,5 μm — PM2,5 (Monitores Fixos e Sistema de Informações Ambientais Integrado à Saúde Ambiental — SISAM). Salvador (BA), 2011 a 2016.

*%RR: (risco relativo −1)*100

As curvas de exposição-lag-resposta para os efeitos acumulados do PM_2,5_ ao longo de 30 dias indicaram baixo risco de morte nos primeiros dias após a exposição, com maior incerteza nos resultados derivados dos dados calibrados do SISAM em comparação com os dados dos monitores fixos (Figura 6). O coeficiente de concordância entre as estimativas de RR dos dados medidos e dos dados calibrados do SISAM foi de 0,80 (IC95% 0,70–0,88), indicando boa concordância entre as medidas de risco.

**Figura 6:** Risco relativo (RR) (intervalo de Confiança de 95%) acumulado de óbitos associado ao aumento 10 μg/m^3^ nos níveis de material particulado com diâmetro inferior a 2,5 μm — PM2,5 (Monitores Fixos, Sistema de Informações Ambientais Integrado à Saúde Ambiental — SISAM Calibrado). Salvador (BA), 2011 a 2016.

Estruturas de *lag* cumulativas do lag 0 ao lag 30, com restrição de um polinômio de segundo grau. A área de sombra representa IC95%.

Como a maioria dos RR estimados não foi estatisticamente significativa, não é possível afirmar que a exposição ao PM2,5 seja fator de risco para a mortalidade em Salvador no período estudado.

## DISCUSSÃO

Os resultados deste estudo demonstram a subestimação significativa dos níveis de PM2,5 modelados por sensoriamento remoto e revelam a necessidade de calibração para garantir a acurácia dos dados nos estudos de avaliação de risco na saúde. Apesar do viés sistemático e dos erros heterocedásticos identificados, o método de calibração simples proposto ajustou efetivamente as estimativas de PM2,5 do SISAM, demonstrando o potencial dos dados modelados por sensoriamento remoto para fornecer informações valiosas sobre a exposição à poluição do ar e suas implicações para a saúde. Destaca-se a situação crítica da poluição do ar em Salvador, com os níveis de PM2,5 excedendo as diretrizes da OMS^39^.

Embora a concordância entre os dados médios dos monitores fixos e do SISAM tenha sido baixa, os efeitos estimados pelos modelos de regressão apresentaram alta concordância, e as diferenças entre as estimativas de risco relativo não foram estatisticamente significativas. Esses resultados sugerem que os dados provenientes do SISAM podem ser uma fonte confiável para avaliar os efeitos da poluição atmosférica na saúde, especialmente porque a calibração da série temporal não alterou a tendência, a sazonalidade e a variabilidade da série.

A aplicação de dados modelados na avaliação da exposição à poluição atmosférica para estudos de saúde é um campo em expansão dentro da epidemiologia^40–42^. No entanto, a utilização de dados modelados sobre a qualidade do ar sem a devida e específica validação para o local de estudo pode levar a subestimações ou sobrestimações dos impactos da poluição atmosférica na saúde. Existem diferentes métodos de calibração para dados modelados, como o proposto neste estudo, que efetivamente corrigiu a distribuição dos dados modelados do SISAM^43,44^.

Embora boa parte dos riscos estimados tenha sido não significativa, a exposição diária a baixas concentrações de poluentes representa em longo prazo uma exposição acumulada que pode impactar a qualidade de vida^45–48^. Estudos epidemiológicos de séries temporais têm sido usados para quantificar os riscos associados à exposição de curto prazo a poluentes atmosféricos na saúde^13–17^. Um estudo em São Paulo observou que os poluentes PM10, NO_2_ e CO foram significativamente associados a mortes não acidentais e por causas específicas tanto nos modelos de defasagem de um dia (*Single Lag*) quanto nos modelos de defasagem acumulada^16^. Para a mortalidade não acidental, em Salvador, o risco estimado associado à exposição ao PM2,5 foi de 0,5% (IC95% −2,1–3,3) e, na cidade de São Paulo, foi de 0,6% (IC95% 0,4–0,8%).

É importante destacar a plataforma SISAM porque ela opera segundo princípios de dados abertos. Esses dados contribuem para o monitoramento da qualidade do ar, a comunicação de riscos e a investigação sobre os impactos da poluição atmosférica nos resultados de saúde. A comunicação de risco no Brasil tem como base a Resolução do Conselho Nacional do Meio Ambiente (CONAMA) n° 491, que tem como referência os valores guia de qualidade do ar recomendados pela OMS em 2005, que indica como padrão para a exposição diária ao PM2,5 o valor de 25 μg/m^3^. Essa concentração foi revisada e atualizada pela OMS em 2021 para o valor de exposição diária de 15 μg/m^3^. Para o período estudado em Salvador, o valor médio de PM2,5 corrigido foi de 12,5 μg/m^3^ e o valor máximo de 31,2 μg/m^3^. O valor máximo ultrapassou duas vezes o limite da OMS, o que aponta um estado de alerta para determinados bairros de Salvador, considerando-se os grupos vulneráveis como gestantes, crianças menores de cinco anos e pessoas com comorbidades.

Vale destacar que o estudo de caso do município de Salvador é uma aplicação local, caracterizada por condições meteorológicas estáveis e baixa variabilidade das concentrações de PM2,5, fatores que contribuem para resultados melhores de dados modelados por satélites para avaliações da qualidade do ar na região.

Embora essa ferramenta de dados modelados por satélites seja usada nos Estados Unidos desde 2009 em estudos de saúde ambiental^23^, algumas lacunas devem ser discutidas, como por exemplo:

1. As concentrações de material particulado ao nível do solo são medidas pontuais em locais fixos, que podem ser mais representativas da zona de respiração em comparação com o valor bruto de profundidade óptica do aerossol (AOD), que representa a integração de toda a coluna atmosférica;
2. As concentrações de material particulado no solo são monitoradas continuamente (por exemplo, hora em hora, a cada três horas por um período de 24 horas), enquanto o AOD é medido quando o satélite passa, que é normalmente uma vez por dia para os instrumentos usados com mais frequência; por isso a medida derivada do satélite não necessariamente representa a variabilidade em cada local;
3. Para os estudos de saúde é importante conhecer a composição do material particulado, porque isso auxilia na compreensão da toxicidade para a saúde humana, e o dado modelado não permite esse conhecimento;
4. Os dados de satélite são sub-representados em dias com elevada nebulosidade, pois esta mascara as habilidades de recuperação dos dados.

Nesse sentido, Salvador é cidade privilegiada, porque apresenta baixa variabilidade dos parâmetros meteorológicos que podem interferir nas medidas de AOD e sua modelagem. Os dados da plataforma SISAM para o município de Salvador são válidos para estimar os riscos à saúde da exposição ao PM2,5 por meio de modelos de séries temporais, embora subestimem a concentração média diária do poluente. Assim, este estudo destaca a subestimação dos níveis de PM2,5 pelo SISAM e recomenda a aplicação de um fator de correção de 5 μg/m^3^ para melhorar a acurácia. Embora existam as incertezas inerentes a dados modelados, como é o caso do SISAM, ressalta-se que a plataforma ainda se constitui como uma das melhores para estudos da qualidade do ar quando não é possível o monitoramento contínuo com técnicas de amostragem avançadas.
